# An Effective Multifactor Authentication Mechanism Based on Combiners of Hash Function over Internet of Things

**DOI:** 10.3390/s19173663

**Published:** 2019-08-23

**Authors:** Adel Ali Ahmed, Waleed Ali Ahmed

**Affiliations:** Faculty of Computing and Information Technology, King Abdulaziz University, Rabigh, Jeddah 25729, Saudi Arabia

**Keywords:** IoT, authentication, hash function

## Abstract

Internet of Thing (IoT) is the most emerging technology in which all the objects in the real world can use the Internet to communicate with each other as parts of a single unified system. This eventually leads to the development of many smart applications such as smart cities, smart homes, smart healthcare, smart transportation, etc. Due to the fact that the IoT devices have limited resources, the cybersecurity approaches that relied on complex and long processing cryptography are not a good fit for these constrained devices. Moreover, the current IoT systems experience critical security vulnerabilities that include identifying which devices were affected, what data or services were accessed or compromised, and which users were impacted. The cybersecurity challenge in IoT systems is to find a solution for handling the identity of the user, things/objects and devices in a secure manner. This paper proposes an effective multifactor authentication (CMA) solution based on robust combiners of the hash functions implemented in the IoT devices. The proposed CMA solution mitigates the authentication vulnerabilities of IoT and defends against several types of attacks. Also, it achieves multi-property robustness and preserves the collision-resistance, the pseudo-randomness, the message authentication code, and the one-wayness. It also ensures the integrity, authenticity and availability of sensed data for the legitimate IoT devices. The simulation results show that CMA outperforms the TOTP in term of the authentication failure rate. Moreover, the evaluation of CMA shows an acceptable QoS measurement in terms of computation time overhead, throughput, and packet loss ratio.

## 1. Introduction

**Figure 1:**
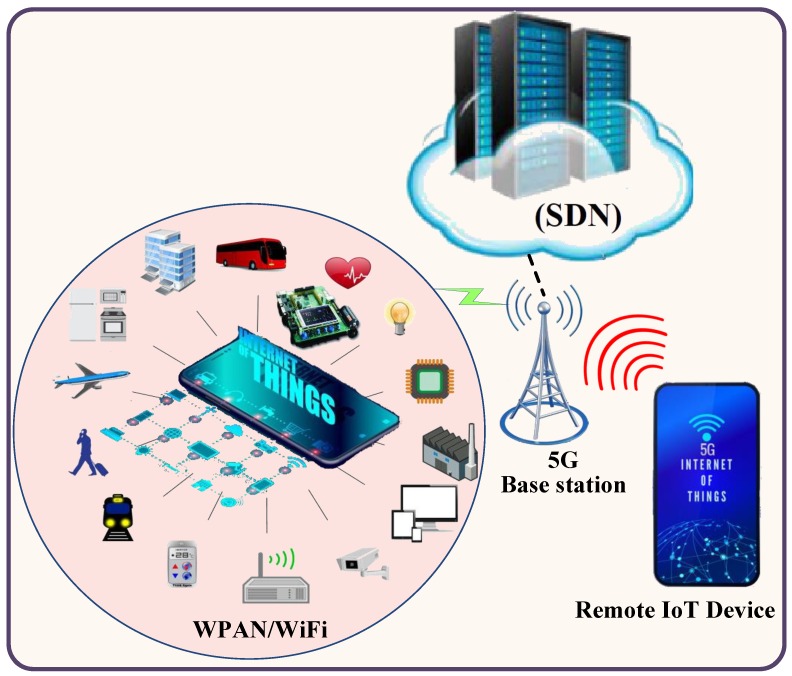
Emergence of the Internet of Things (IoT) architecture.

The explosive growth of wireless network technology has led to scientific challenges, notably in terms of managing the communications between objects and infrastructures without human intervention. One of the most interesting wireless network technologies is the Internet of Things (IoT) which allows IP connectivity and data gathering for a network of devices without human interference. In general, IoT devices are extremely heterogeneous and differ in term of connectivity interfaces, battery, processing and memory capabilities, as well as in dimensions, costs, and hardware features [1]. The emergence of the IoT architecture relies on two types of communication interfaces which are the micro IoT paradigm based on short-range radio technologies (e.g., IEEE 802.15.4/RFID/NFC/IEEE 802.11), and the rising macro IoT paradigm, based on 3G/4G/5G technologies [1,2]. As shown in Figure 1, the multipurpose sensor nodes in the event area are integrated into the electronics and objects to produce the sensor network platform. The sensor network could be combined into the medical equipment in a hospital, household appliances, smart monitoring and controlling devices in the city, etc. Moreover, the sensed information can be sent to the sink node and stored at the base station of a local network or might be forwarded directly to an IoT device.

The IoT device based on 3G/4G/5G can receive the requested information from the sink or directly from the sensor network devices. Furthermore, the 5G base station is used to bridge the IoT network to another network or platform using software defined network (SDN) technology. The SDN offers a logical centralized and programmable method of IoT networks that resolve the weaknesses of traditional networks, such as troubleshooting and reconfiguration of connection for all devices in IoT, effective usage of network resources, reducing latency due to a distributed mechanism, etc. [3,4,5,6]. 

The IoT cyberattacks mean an attempt to damage, disrupt, or gain unauthorized access to any IoT devices (sensor device, base station/sink, or remote IoT). Generally, cyberattacks aimed at accessing, changing, or destroying sensitive information; extorting money from users; or interrupting normal business processes which may outweigh the IoT benefits. Despite the architectural design of IoT, the network model of IoT is vulnerable to several types of cyberattacks at three layers (the application layer, the network layer, and the sensing layer). The IoT cyberattacks might include man-in-the-middle, denial of service (DoS), replay, link spoofing, forced delay, session hijacking, cross-site scripting, and SQL injection attacks [7,8]. 

The main challenges of an IoT network are the lack of cybersecurity standards that can countermeasure the aforementioned cyberattacks. Furthermore, the IoT based on a cryptosystem requires imperative computation overhead for resource-constrained devices because of the exponentiation operations to be executed in the encryption and decryption phases. Thus, IoT networks should implement lightweight authentication mechanism to prevent the unauthorized devices and to verify the access to the IoT services. An effective authentication solution determines if upon identification, the person or device is permitted to receive a service [9,10]. In fact, the authentication issue is critical in IoT, because multiple users, object/things and devices need to authenticate each other through trustable services. Most of the authentication mechanisms are built from the hash function which is employed in a broad spectrum of cryptographic protocols, such as message authentication codes, digital signatures, encryption schemes and key-agreement in the TLS/SSL protocols. An independent approach to achieve hash constructions that are more tolerant to cryptanalytic results is to use so-called combiners. That is, combining multiple (hash) functions in such a way that the resulting function remains secure as long as at least one of the underlying candidates is secure [11,12].

### 1.1. Problem Statement 

Since IoT devices have limited resources, the cybersecurity approaches that relied on complex and long processing cryptography are not a good fit for these constrained devices, especially when those approaches are applied to protect real-time applications [13]. The cybersecurity challenge in an IoT system is to find a solution for handling the identity of the user, things/objects and devices in a secure manner. In addition, the current IoT systems experience critical security vulnerabilities that include identifying which devices were affected, what data or services were accessed or compromised, and which users were impacted, and then taking action to resolve those situations. The fast, lightweight hash algorithms with multiple layers of defense can provide an effective multifactor authentication solution that countermeasures the cyberattacks over IoT networks. The aim of this paper is to provide an effective multifactor authentication solution that tackles the aforementioned problems over IoT Network.

### 1.2. Summary of Contributions

This paper reports the following contributions. Firstly, it proposes an effective multifactor authentication mechanism that uses robust combiners of fast hash functions. Each hash function is calculated based on preset key and the idea of one-time password (OTP). Also, the hash function is applied to both the key and the message. Secondly, it proposes a time-enhanced-based one-time password (TEOTP) hash function that is implemented in the base station/sink and IoT device to resolve the problem of time synchronization in time-based one-time password (TOTP). The time margin is caused by the clock skews, network latency and user delays. Therefore, the proposed TEOTP uses dynamic time synchronization based on round-trip time (RTT) to resolve the time margin problem. Finally, the proposed multifactor authentication mechanism has the capability to manage the direct access control of the IoT device to communicate with the sensor objects directly without intervention of the sink. It uses the concept of open authentication (OAuth) and secure the token credential session period, which will speed up the transfer of sensed data to the IoT device. Moreover, the proposed multifactor authentication (CMA) guarantees the integrity, authenticity and availability of sensed data for the legitimate IoT devices.

The paper is structured as follows: Section 2 presents the related works on IoT cyberattacks solutions. The system design of the proposed cybersecurity mechanism is explained in Section 3. Section 4 describes the cryptanalysis for the proposed cybersecurity mechanisms. Section 5 explains the performance analysis and the discussion of the obtained results. Also, Section 6 describes the potential limitation for implementing CMA. Finally, Section 7 concludes the paper.

## 2. Related Work on IoT Cybersecurity

Many researchers have studied the IoT cybersecurity from layer-level perspectives; however, some cyberattacks appear at most layers of the IoT network [14]. The related works in this paper focus on the research studies that concern the cyberattacks and countermeasures to the entire IoT network. 

Cirani et al. [15] proposed an external OAuth-based authorization service, denoted as IoT-OAS, which provides HTTP and CoAP service providers with an authorization layer to be able to disseminate their services without the need for implementing the OAuth logic. Hummen et al. [16] proposed a delegation architecture that offloads the expensive DTLS connection establishment to a center delegation server. The delegation architecture in [16] relied on the certificate-based DTLS handshake protocol which is the main IP security solution for IoT. However, their proposed architecture suffers from a considerable network transmission overhead resulting in a long transmission latency. Furthermore, Moosavi et al. [17] developed a secure and efficient authentication and authorization architecture for IoT-based healthcare systems using distributed smart e-health gateways. The research presented in Aman et al. [18] proposed an efficient protocol for mutual authentication in IoT systems which uses a physical unclonable function (PUF) based on a challenge–response mechanism. The most related research is presented in Aman et al. [19] which proposed a location-based authentication protocol for IoT systems. The authors used two-factor authentication which depends on PUFs and the current location of an IoT node within a circular area. Also, Gope et al. [20] proposed a lightweight and privacy-preserving two-factor authentication protocol for IoT devices where physically unclonable functions have been considered as one of the authentication factors. Furthermore, Li et al. [21] proposed a lightweight mutual authentication protocol based on a novel public key encryption scheme for smart city applications. Also, Sciancalepore et al. [22] proposed a key management protocol (KMP) which suitably integrates implicit certificates with a standard elliptic curve Diffie–Hellman exchange, and performs authentication and key derivation. The authors also provided peers’ authentication, ephemeral key derivation, fast rekeying, and efficient protection against replay attacks. The research presented in Xiong et al. [23] proposed a three-factor anonymous authentication scheme for WSNs in IoT environments, where the fuzzy commitment scheme is adopted to handle the user’s biometric information. Kumari et al. [24] proposed an authentication scheme based on elliptic curve cryptography (ECC) for IoT and cloud servers. Also, Dhillon et al. [25] proposed ECC-based authentication protocol based on cloud-IoT environments to monitor remote patient in real-time. Moreover, Xie et al. [26] proposed a dynamic ID-based anonymous two-factor authenticated key exchange (AKE) protocol, which addressed lost-smart-card attack, offline dictionary attack, de-synchronization attack. It supported the smart card revocation and password update without centralized storage. Furthermore, Chatterjee et al. [27] proposed a deep neural network-based framework that allows real-time authentication of IoT devices using the effects of inherent process variation on RF properties of the wireless transmitters (Tx), detected through in-situ machine learning at the receiver (Rx) end. The research presented in Alizai et al. [28] proposed a secure and efficient multi-factor device authentication scheme that uses digital signatures and device capability to authenticate a device. Also, Shah et al. [29] developed a multifactor authentication system which used exclusive-or operations, encryption algorithms and Diffie–Hellman key exchange algorithm to share key over the network.

The limitations of previous literature studies [15,16,17,18,19,20,21,22,23,24,25,26,27,28,29] are basically divided into three points: Firstly, most of the research studies implemented the authentication mechanism on the wireless sensor networks while the outstanding architecture of IoT is not considered. Secondly, the direct access between the IoT devices and the sensor devices was not investigated. Finally, the discrepancy of IoT devices’ capabilities was not considered in the design of the authentication mechanism. 

## 3. System Design of Proposed Cybersecurity Mechanism

The proposed multifactor authentication algorithm presents three scenarios that cover the main important authentication cases in IoT network. The authentication scenarios that will be described in this section comprise of the communication between an IoT device and the base station (scenario1); the communication between an IoT devices and sensor device through the base station (scenario2); and the direct communication between IoT devices and sensor device (scenario3). 

### 3.1. Multifactor Authentication Algorithm

The user ID and password authentication mechanism are the most classical method among authentication techniques on the internet; however, it is a vulnerable method against eavesdropping or replay attacks. The multifactor authentication algorithm creates a unique one-way digital fingerprint that represents the contents of IoT packets. In order to cope with the three aforementioned scenarios, this paper proposed three authentication algorithms, which are micro IoT paradigm authentication, macro IoT paradigm authentication, and micro–macro paradigm authentication. These are designed based on the following assumptions:

- Each sensor device has three secure keys—two privates (K1 and K2) and one public (K_DSA: key for direct access control)—which are stored during device programming.
- Each sensor device static or mobile is aware of its location.
- Sink is a trusted base station.
- A sensor device cannot use TOTP because it has limited resources which affect the precision calculation of the absolute time that is required in a synchronous TOTP.
- Each IoT device has two secure keys—one private (K_I, ID_) and one public K_DSA.
- An IoT device and the sink have an ability to implement TOTP and the TEOTP.
- A sink or a base station has a database that stores the complete details of all sensors and IoT devices.

The following subsections describe the three algorithms and explain how these algorithms operated on IoT networks.

#### 3.1.1. Micro IoT Authentication Paradigm

**Figure 2:**
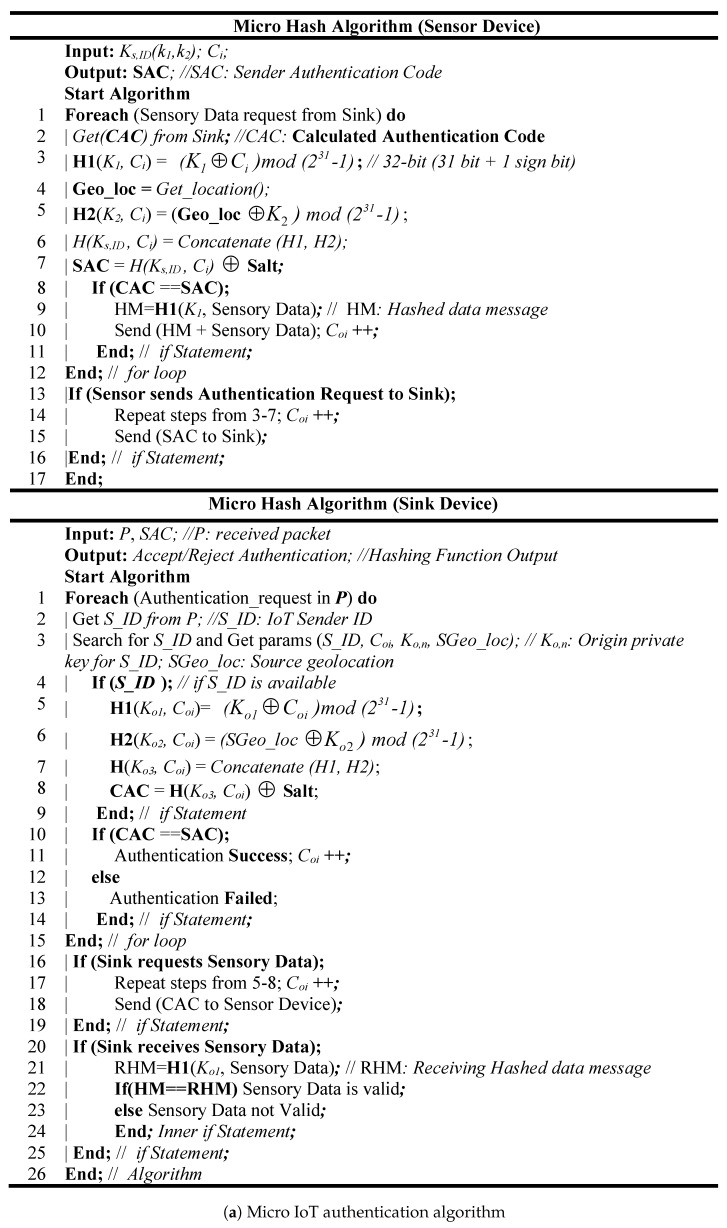

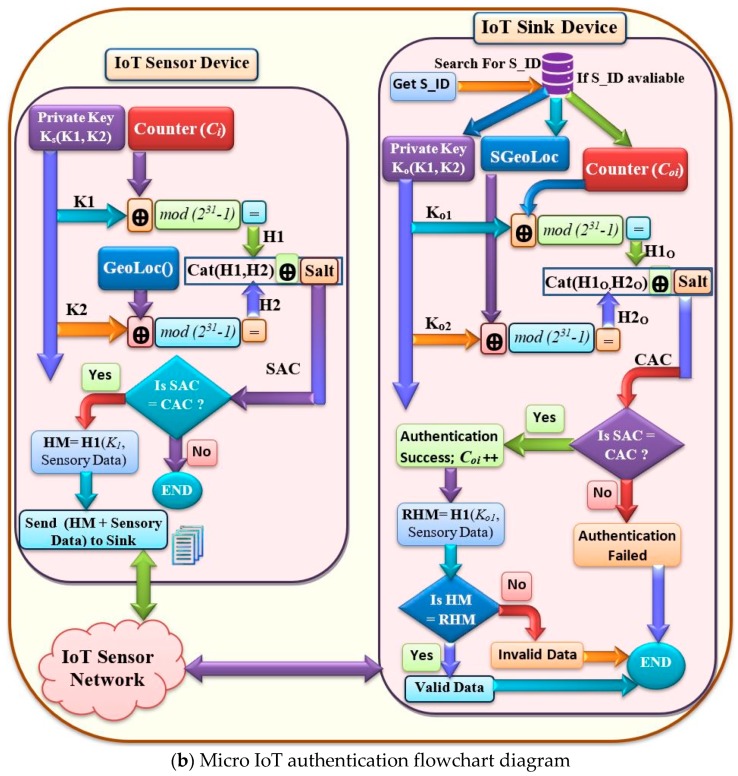
System design of Micro IoT authentication.

The micro IoT authentication paradigm is developed based on two authentication credentials which are “WHAT YOU KNOW: Private Key” and “WHERE YOU ARE: Geolocation” that verify the genuineness of the sensor nodes and the sink. We assume that each sensor node has two private keys and a counter number synchronized with the legitimate sink. The first private key is ***K_1_*** which is used with the first authentication credential, and the second is ***K_2_*** which is used with the second authentication credential. Both keys (***K_1_***, ***K_2_***) and the initial value of the counter number ***C_i_*** are uploaded into the sensor device during programming. Figure 2a,b shows the pseudo code and the flowchart diagram of the micro hash algorithm. In this algorithm, if the legitimate sink requests sensed data from any sensor device (let us assume sensor device ***A***), the calculated authentication code (CAC) should be sent from the sink to sensor device ***A*** which will be used to verify the authorization of the sink. In order to validate the CAC at sensor device ***A***, the first factor authentication is calculated based on a first hash function ***H1*** which will use ***K_1_*** and the last value of ***C_i_***. In addition, the sensor device ***A*** will calculate the second factor authentication based on the second hash function ***H2*** which will use the geolocation of sensor devices ***A*** and ***K_2_***. After that, the sender authentication code (SAC) is calculated based on combiners of both hash functions, and SALT random string as can be expressed in the following equation:

(1) Comb(H1,H2,SALT)=(H1(K1⊕C)||H2(K2⊕L))⊕SALT

where ***L*** is the current location of sensor device ***A*** which will increase the strength of hashed code. Moreover, the SALT consists of a uniform random string which is used to defend against the password attack. Also, Equation (1) shows that the maximum output length of the combiner is 62 bytes. The SAC is compared with the CAC; if they are matched, then the sink is authorized. Otherwise the sink authentication is failed. Furthermore, the sensor device ***A*** will use ***H1*** to hash the sensed data and send both the original message and the output of ***H1*** to the sink. Upon receiving the authenticated message, the sink will recalculate the hash of receiving data. If the received hash code is matched with calculated code, the sink will accept the authenticated message. Otherwise, the sink will discard the received message. Moreover, the proposed micro IoT authentication is a mutual authentication which means if the sensor device ***A*** periodically sends the hashed sensed data to the legitimate sink, the sink will not accept the sensed data unless the calculated hash message code is matched with the received hash code.

#### 3.1.2. Macro IoT Authentication Paradigm

**Figure 3:**
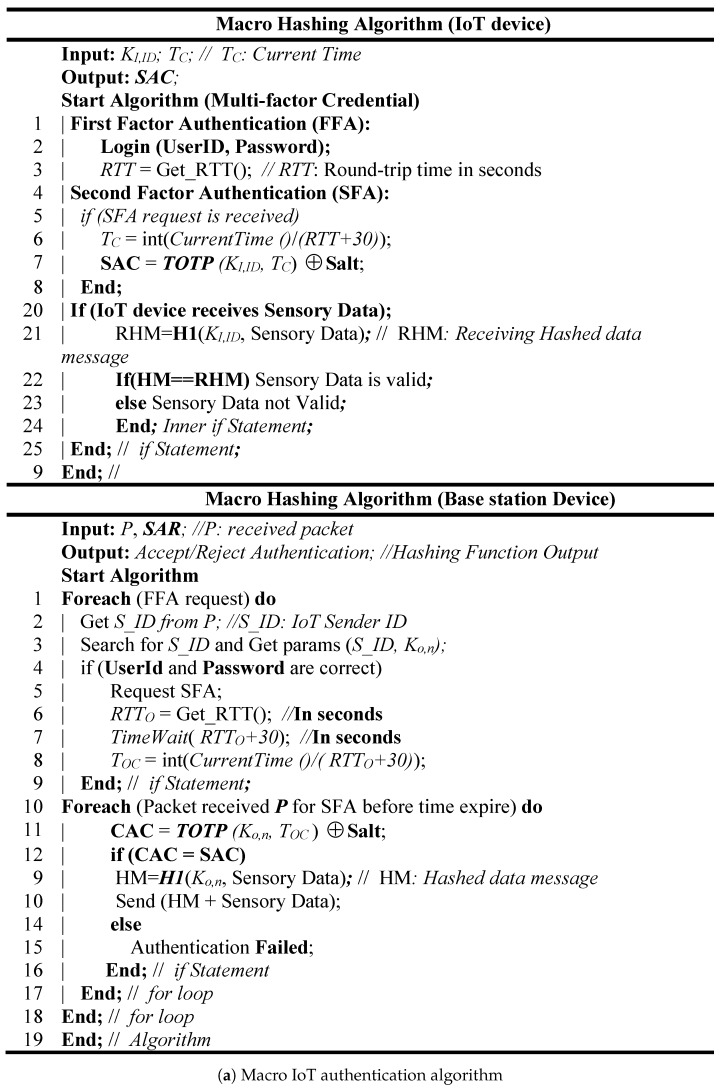

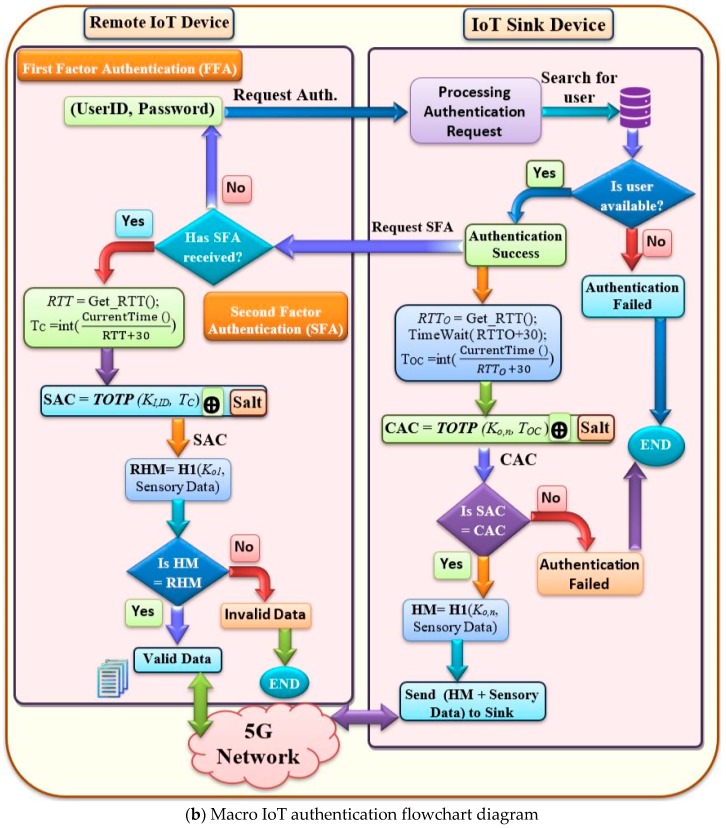
System design of Macro IoT authentication.

The macro IoT authentication paradigm is developed based on the multifactor authentication that used “WHAT YOU KNOW: Passwords” and “WHAT YOU HAVE: Token” authentication credentials to verify the genuineness of the IoT device and the sink. We assume that each legitimate IoT device has a login information (UserID, Password) and a counter number synchronized with the legitimate base station. Figure 3a,b shows the pseudo code and the flowchart diagram of the micro hash algorithm. In this algorithm, the first factor authentication (FFA) at the legitimate base station is verified based on user ID and password of the IoT device. Moreover, the second factor authentication (SFA) is verified based on an enhanced algorithm of time-based one-time password which called TEOTP. In SFA, the current time which is also used in TOTP is a UNIX time (UNIX Epoch time) that is calculated based on the number of seconds that have elapsed since 00:00:00 Thursday, 1 January 1970. Due to the latency of data transmission between the IoT device and the base station, TOTP must validate over a range of times between the IoT device and the base station. In TOTP, the time is down-sampled into interval durations (e.g., 30 s) to allow for validity between the parties. However, the interval duration in TOTP is static and vulnerable to clock skews, network latency and user delays between the base station/sink, and IoT device. TEOTP resolves the static interval of time synchronization problem using dynamic interval durations based on round-trip time (RTT) which is added to the 30 s. Equation (2) describes the combiner of TEOTP and SALT random string as follows:

(2) $$Comb(H1,SALT)=TOTP(K1,\frac{Time()}{30+RTT})⊕SALT$$

The main advantage of RTT is to regulate the interval durations which will resolve the delay variation due IoT network congestion or forced delay attacks. After that, SAC is calculated based on the output code of TEOTP which is sent to the base station and is compared with the CAC. If SAC and CAC are matched, then the IoT device is authenticated and the requested data is sent; otherwise the IoT device authentication operation fails. Like the micro IoT authentication algorithm, the macro authentication is a mutual authentication and it is applied to both the key and the message. This means if the base station/sink sends certain data to the legitimate IoT device, the IoT device will not accept these data, unless the calculated hash message code is matched with the received hashed message code.

#### 3.1.3. Micro–Macro IoT Authentication Paradigm

**Figure 4:**
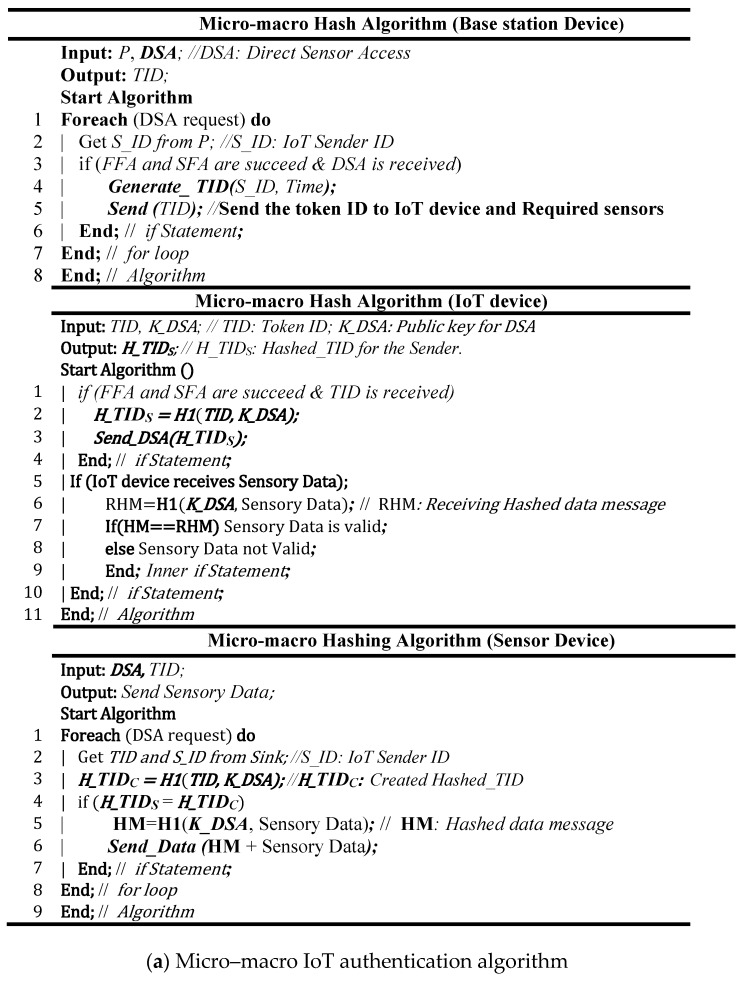

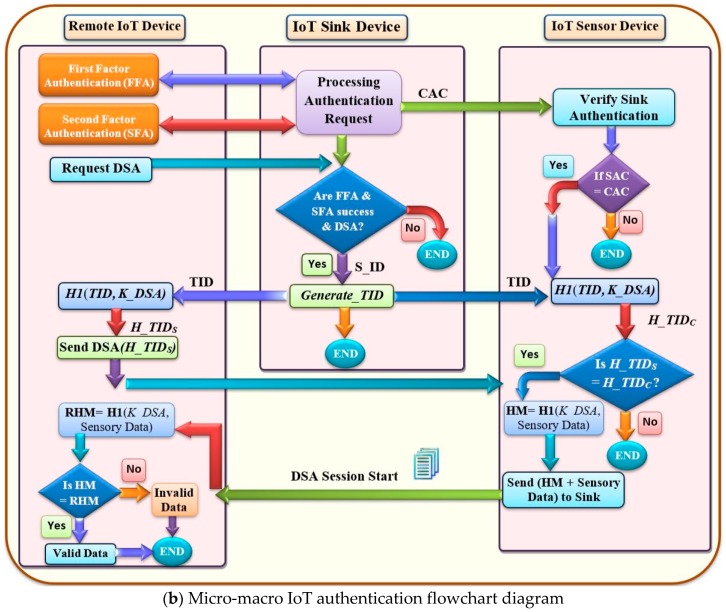
System design of micro–macro IoT authentication.

The micro–macro IoT authentication paradigm is aimed to allow IoT devices to communicate with the sensor device without intervention of the base station or the sink. The open authentication (OAuth) concept has been used to develop the micro–macro IoT authentication algorithm. OAuth permits sensor devices to share sensed data with an IoT device without intervention of the base station. At the initial state, the base station uses the OAuth mechanism to forward the token credentials to both the IoT and sensor devices which will use the hashed token ID to verify each other. Figure 4a,b shows the pseudo code and the flowchart diagram of the micro–macro hash algorithm. In this algorithm, we assume that the IoT device requests the base station to access the sensor device directly without intervention of the base station. This mode of IoT operations is called direct sensor access (DSA). After the IoT device is authenticated, the base station/sink will send secure token credentials to the sensor device(s) and the IoT device as well for a limited period of time. The IoT device should encrypt the Token ID (TID) using H1 hash function and it sends the hashed token ID (H_TID) to the all sensor devices that involved in DSA. The secure key that is used in H1 hash function is replaced with public key called K_DSA. Also, the TID works as the counter number *Ci* in the micro IoT authentication algorithm. If the transmitted H_TIDS and the created H_TIDC by the sensor device are matched, then the IoT device is authenticated and the DSA session is started; otherwise the IoT device authentication operation fails. Like the micro and the macro IoT authentication algorithms, the micro-macro authentication is a mutual authentication and it is applied to both the key and the message. This means if the sensor device sends certain data to the legitimate IoT device, the IoT device will not accept these data, unless the H_TIDS and the received hash message code are matched with the H_TIDC and the calculated hash message code.

## 4. Cybersecurity Analysis

**Figure 5:**
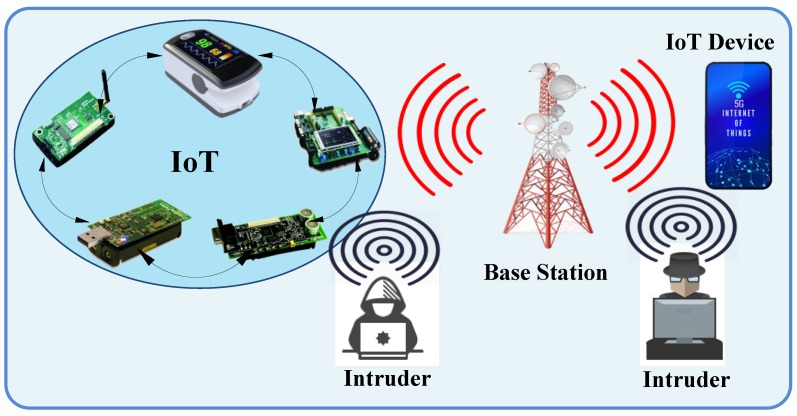
Structure diagram of possible cyberattacks in IoT.

The basic cybersecurity problem in the IoT is that the attackers can gain access to the sensitive information that are restricted from obtaining by the third party. This section will explain how the proposed CMA mechanism can reduce the authentication vulnerabilities in IoT and how it defends against several types of attacks such as replay attack, link spoofing attack, man-in-the-middle attack, dictionary attack, brute force attack, sensor capture attack, stolen-verifier attack, and session hijacking attack. Figure 5 depicts the structure diagram of possible cyberattacks in IoT. In this figure, the IoT network is vulnerable to different types of attacks by intruders which can target the communication between sensor devices, the communication between the sensors and the sink/IoT devices, and the communication between the base station and IoT devices.

### 4.1. Multi-Property Robustness of the Proposed Mechanism

The CMA underlying hash functions achieves multi-property robustness (MPR) which means it should simultaneously preserve the collision-resistance (CR), the pseudo-randomness (PRF), the message authentication code (MAC), and one-wayness (OW). For multiple properties PROP = {CR, PRF, MAC, OW} one can either demand that the proposed combiner inherits the properties if one of the candidate hash functions is strong and has all the properties (weakly robust), or that for each property at least one of the three hash functions has the property (strongly robust). We denote by PROP(H) ⊆ PROP for a set prop = PROP = {CR, PRF, MAC, OW} the properties which a hash function H has. In order to show that the proposed combiners of hash functions satisfy the strongest notion of MPR, the following mathematical proof is described as follows:

**Theorem** **1.** *The proposed combiner in Equations (1) and (2) is a strong MPR combiner for PROP = {CR, PRF, MAC, OW}.*

**Proof.**  As we have mentioned that a strongly robust multi-property combiner should inherit all properties that are provided by at least one of the underlying hash functions. Thus, we have to prove that each property CR, PRF, MAC and OW is preserved individually. □

**Lemma** **1.** *The proposed combiner in Equations (1) and (2) is CR-robust.*

**Proof.**  In CR property, it should be hard to find two distinct inputs that evaluate to the same hash value. The proof of CR property in CMA can be basically observed in H1 (***K1***, ***C_i_***) and in TEOTP (***K1***, ***Time***) in which the value of counter number ***C_i_*** and ***Time*** are distinct for all inputs. This is mainly because the value of ***C_i_*** and ***Time*** is continuously incremented after each authentication operation and the distinctness of the combiner of ***K1*** ⊕ ***C_i_*** and TEOTP (***K1***, ***Time***) is also preserved for all inputs. Hence, the expected time for the CR in the proposed combiner is O(2^n^) where *n* is 62 bits in Equation (1) and 31 bits in Equation (2). □

**Lemma** **2.** *The proposed combiner in Equations (1) and (2) is PRF-robust.*

**Proof.**  In PRF property, the combiner is called pseudorandom if no efficient adversary can distinguish the output of the combiner from a uniform random function with noticeable advantage. Since the proposed combiner xors the SALT (a uniform random function) with the concatenation of (H1, H2) in Equation (1) and with TEOTP in Equation (2), the final output of proposed combiner preserves the PRF property. Hence, the final output is indistinguishable from the uniform random and is PRF robust. □

**Lemma** **3.** *The proposed combiner in Equations (1) and (2) is MAC-robust.*

**Proof.**  The CMA used a MAC concept in the three proposed algorithms which allows a sender and receiver, both sharing a secret, to exchange information in an authenticated manner. A MAC is considered secure if it is unforgeable under chosen message attacks, i.e., an adversary after adaptively learning several tags (M1, τ1), (M2, τ2),…, (Mq, τq) should not be able to compute a forgery for a fresh message M. In CMA, MAC is calculated based on xoring of a secret K1 with the data message (M). Furthermore, the strong condition in MAC calculation is that the SAC should be verified between the sender and the receiver before evaluating the MAC. Therefore, even if the adversary creates a forgery message M, it will not be accepted because the verification of SAC is not known by the adversary. □

**Lemma** **4.** *The proposed combiner in Equations (1) and (2) is OW-robust.*

**Proof.**  In OW-robust, the proposed combiner intuitively requires that it is infeasible to determine the preimage of a hash value. Since H1 and H2 in Equations (1) and (2) use the modulo operation with 2^31^, it is hard to invert the H1 and H2 for longer input length then 2^31^. Moreover, the input length of ***C_i_*** and ***Time*** is continuously incremented, and the SALT also increases the input length of the proposed combiner to reach the maximum value with 2^62^. Therefore, the probability for the adversary to determine the preimage of the combiner output is 1/2^62^ which means the OW is preserved in CMA. □

### 4.2. Countermeasures of the Proposed Mechanism

The proposed cybersecurity mechanism ensures three security requirements which are authenticity, integrity, and an availability of sensed data. The authenticity of the legitimate sink and IoT devices is achieved using the multifactor authentication which does not allow the intruders to gain access to the IoT network. Moreover, the SALT which consists of a random string is used to defend against the password attack. In addition, the proposed cybersecurity mechanism provides seamless integrity for the sensed data that is transferred between the IoT devices. This is primarily due to the proposed hash function is applied to both the authentication code and the message which can detect the alteration of sensed data when it is transferring between IoT devices. Also, it guarantees the availability of sensed data to the authorized IoT devices. This is mainly due to the proposed cybersecurity mechanism does not allow the intruders to associate with the IoT network and make flooding/congestion attacks.

#### 4.2.1. Countermeasures against Man-in-the-Middle and Replay Attacks

A man-in-the-middle attack intercepts the legitimate communication between IoT devices, and it forges a fictitious response to the sender. In an active man-in-the-middle attack, the contents are intercepted and altered before they are sent on to the recipient [8]. Moreover, a replay attack is similar to a man-in-the-middle attack. Instead of sending the transmission immediately, a replay attack makes a copy of the transmission before sending it to the recipient. This copy is then used later. However, using the proposed CMA mechanism, man-in-the-middle and replay attacks can intercept the communication between IoT devices, but he/she cannot forge a fictitious response to the sender who will not accept the reply message from those attacks due to the following reasons:

- The multifactor credentials should be inspected between a man-in-the-middle and the sender before accepting any data message.
- The reply message should be hashed using the secret key which is not included in the original message and only known by the sender and the base station.
- The replay attacks cannot forward the copy of sending messages, because the proposed CMA uses TEOTP which changes after a set time period (30 s + RTT).

#### 4.2.2. Countermeasures against Dictionary and Brute Force Attacks

A dictionary attack is a password attack that creates encrypted versions of common dictionary words and compares them against those in a stolen password file. Moreover, a brute force attack is a password attack in which every possible combination of letters, numbers, and characters is used to create encrypted passwords that are matched against those in a stolen password file [8]. However, the proposed CMA mechanism can defend against those attacks using the combiners of hash function combined with login information (user Id and password). The combiners of hash function in the CMA are H (Cat (H1, H2), TEOTP, and OAuth which were applied to micro and macro IoT authentication. Therefore, the CMA combines plaintext with a random key which is the only known method to perform encryption that cannot be broken mathematically. In addition, the SALT random string makes dictionary and brute force attacks for cracking many passwords much difficult. Another benefit of the SALT is that if many users choose the same password, this will not help the attacker because the SALT will append a random string to the similar passwords.

#### 4.2.3. Countermeasures against Spoofing and Session Hijacking Attacks

A spoofing attack is impersonating another IoT device in which an intruder spoofs the network address of the target IoT device so that their malicious actions will be attributed to valid IoT devices. Moreover, a session hijacking is an attack in which an intruder attempts to impersonate the IoT device by using its session token identity. However, the proposed CMA mechanism can defend in advance against these attacks by using the multifactor authentication, which uses the combination of geolocation information, OAuth hashed token ID, and TEOTP credential code. Moreover, the limited time of TEOTP credential code will make the session identity code periodically change. Even if the intruder still gains the session ID, he/she cannot gain access to the IoT network because he/she needs to calculate the multifactor of credential code for every sending or receiving message.

#### 4.2.4. Countermeasures against Sensor Capture and Stolen-Verifier Attacks

A sensor node capture attack means that an intruder captures a sensor node, steals all the information stored (keys and measured data) and uses the compromised keys to perform various operations on the IoT network. Moreover, a stolen-verifier attack means that the intruder who has stolen the verifier data for the sensor/IoT device can impersonate a legal device from the next authentication session. However, the proposed CMA mechanism defends against those attacks using the combiners of hash functions that used the one-time synchronize random input based on *Ci* and Time. Moreover, the embedded hash functions in the execution file of the source code will prevent the node capture attack to gain access to the IoT network because those functions are uploaded into the sensor device using machine language code. Furthermore, the requirement of geolocation information which depends on the actual physical location of sensor devices will restrict the effect of the captured sensor node. Also, the OAuth mechanism can prevent the stolen-verifier attack because the one-way hash function is implemented at the trust base station and the intruder cannot verifies the hashed token ID.

## 5. Implementation of CMA and Evaluation

**Figure 6:**
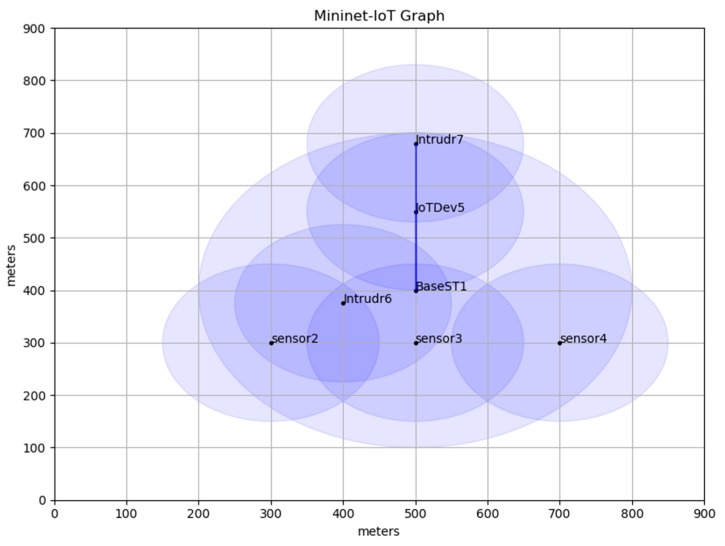
IoT emulation topology.

To demonstrate the CMA functionality, several experiments have been conducted using Mininet-IoT emulation software [30]. A small mesh topology network was comprised of three sensor devices (sensor1 to sensor3), one base station (BaseST1), one IoT device (IoTDev5), and two intruders (Intrudr6 and Intrudr7). Each sensor device can communicate with the base station directly using 6LowPAN protocol. The header size and the maximum transfer unit in 6LowPAN is 40 bytes and 127 bytes respectively [31]. As shown in Figure 6, the IoT device can communicate with the base station using two types of protocol which are IPv6 and 6LowPAN. Moreover, The IoT device can communicate with the sensor devices through the base station or directly using DSA mechanism. To emulate the attack, we assumed Intrudr6 and Intrudr7 were two attackers that could implement any types of attacks. As the network manager does not configure Intrudr6 and Intrudr7 with the IoT network name (SSID), those intruders should not be able to associate themselves with the IoT network; hence, they should not be reachable by all sensors, IoT device, and base station and vice-versa.

Table 1 shows the details about the emulation configuration parameters and setting. In this table, 802.15.4_hwsim and 802.11_hwsim models have been designated to implement a micro and macro environment of the IoT network. Also, the shadowing propagation model has been used to reflect the actual signal degradation due to interference in the propagation path. The IoT topology is emulated based on a grid network area of 1000 m × 1000 m (1.0 Km^2^). The mobility model of IoT devices and intruders is established using random movement on the straight line. Also, the traffic load is measured using the number of packets that are sent per second (pkt/s). The emulation time has been set to 1000 s to give the intruders the enough time to implement dictionary and brute force attacks. The velocity of IoT device was randomly varied between 5 m/s and 15 m/s. Furthermore, the key length size (K1, K2, SALT) is 32 bits and the uniform random function that has been used in the sensor device is simulated based on [32,33].

### 5.1. Performance Evaluation and Results Discussion

In this section, the performance of using the three proposed authentication algorithms—micro, macro, and micro–macro paradigm authentication—has been analyzed in terms of latency, throughput, and packet loss ratio over IoT network. Moreover, the comparison between the proposed TEOTP and TOTP algorithms has been investigated. The throughput can be defined as the amount of data transferred successfully to the destination in a given period. The packet loss ratio is defined as the ratio of the total number of lost packets due to using CMA to the total number of sending packets. The latency is the period between the transmission and the reception of the packet when CMA is applied to an IoT network.

#### 5.1.1. Impact of Proposed Authentication Algorithms on IoT Performance

**Figure 7:**
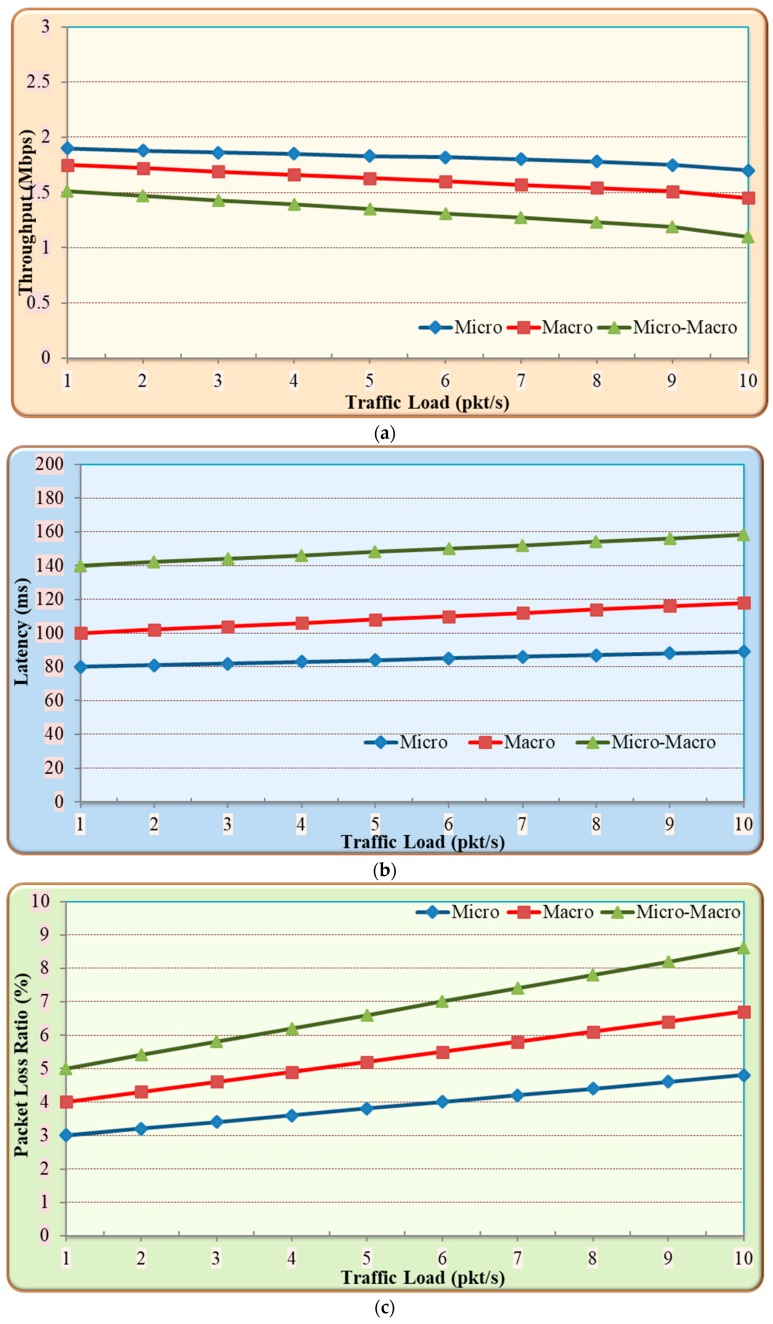
Impact of CMA authentication algorithms on IoT Performance (**a**) throughput; (**b**) latency; (**c**) packet loss ratio.

In this experiment, the effect of using CMA authentication algorithms was evaluated in terms of latency, throughput, and packet loss ratio over IoT network. The Iperf of IPv6 standard tool was used to measure the performance of conducting TCP data traffic tests. In order to create data streams to measure the performance between IoT and sensor devices, the Iperf client function was run in the sensor3 and the Iperf server function was run in BaseST1 for micro authentication experiment. Moreover, the Iperf server function was run in IoTDev5 for macro and micro–macro authentications experiment. Figure 7 shows the performance results of using CMA authentication mechanisms on IoT network. In Figure 7a, the throughput of micro authentication experiences on average 19% higher than the average throughput of macro and micro-macro authentication mechanisms. Moreover, Figure 7b shows the latency of the micro authentication experiences 28% less time latency compared to macro and micro–macro authentication mechanisms. Also, Figure 7c illustrates that the micro authentication experiences 25% less packet loss ratio compared to macro and micro–macro authentication mechanisms.

- **Discussion**

The above results show the performance of the three authentication mechanisms which are mainly achieved due to the following reasons. Firstly, macro and micro–macro authentication mechanisms use embedded TOTP with RTT modification in an authentication decision which improves the cybersecurity, but it decreases the throughput of IoT network. Secondly, the long processing delay of macro and micro–macro authentication increases the latency of authentication. This is primarily due to the two types of multifactor authentication mechanisms that are implemented between the sensors and the IoTDev5. The first type of multifactor authentication is implemented between the sensors and the sink and the second type of multifactor authentication is implemented between the sink and the IoTDev5. Finally, the packet loss ratio is higher at the beginning of macro and micro–macro authentication mechanisms because of the dropping packets at the verification of data credentials that involve in the combiners of hash function. Overall, the cost of developing an unbreakable cybersecurity mechanism in CMA is acceptable and it does not affect the QoS of an IoT network in terms of packet loss ratio, latency, and throughput.

#### 5.1.2. Comparison between the Performance of TEOTP and TOTP

**Figure 8:**
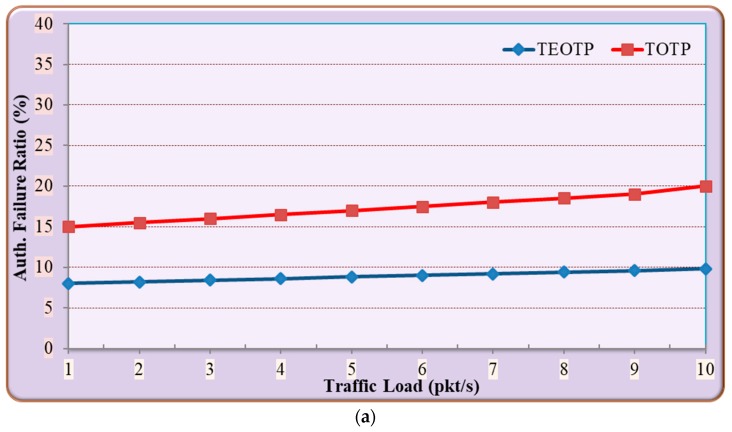

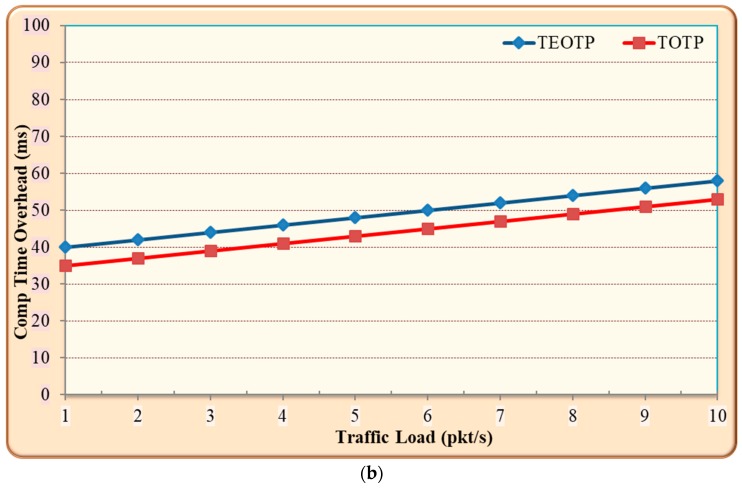
Comparison between the performance of time-enhanced-based one-time password (TEOTP) and time-based one-time password (TOTP): (**a**) authentication failure ratio; (**b**) computation time overhead.

In this experiment, the ICMP and Iperf tool was used to measure the authentication failure rate and computation time overhead based on UDP data traffic tests. In order to emulate the TOTP in Mininet-IoT, the library source code of PyOTP [34] was installed and imported into the macro authentication of CMA source code. The authentication failure ratio is defined as the ratio of failed attempts to the total number of attempts. Figure 8 shows the comparison between the performance of TEOTP and TOTP. In Figure 8a, the TOTP authentication mechanism experiences on average 46% higher authentication failure ratio compared to TEOTP. However, Figure 8b illustrates that the TEOTP experiences on average 12.5% higher computation time overhead compared to TOTP.

- **Discussion**

The above results show that the TEOTP outperforms the TOTP in terms of the authentication failure rate. This achievement is primarily due to the fact that the TOTP authentication mechanism does not handle the network latency between the source and the destination of IoT devices. Moreover, the computation time overhead is higher in TEOTP because of the RTT overhead that is essential to measure the latency between the authenticated parties of IoT devices. More importantly, the RTT involved in TEOTP calculation is used to decrease the authentication failure ratio due to the time synchronization between IoT devices.

## 6. Potential Limitations for Implementing CMA in IoT

Although the CMA was carefully designed for IoT, there were some of unavoidable hardware limitations. Firstly, the CMA cannot be applied to the available IoT network devices due to the limitation of existing hardware and software which means a new generation of IoT network devices should be developed. Secondly, IoT networks scalability and energy consumption need more investigation to cope with the emergence of IoT hardware specifications which yet well-defined in the main standard document of IoT technology. Finally, the proposed CMA software needs advance programming to integrate all authentication mechanisms of CMA together in a sensor/IoT devices.

## 7. Conclusions and Future Work

This paper presents CMA that uses robust combiners of fast hash functions to achieve MPR and preserve CR, PRF, MAC, and OW. Also, it proposes a TEOTP hash function implemented in the base station/sink, and IoT device to resolve the problem of time synchronization in TOTP. The simulation results show that CMA outperforms the TOTP in term of the authentication failure rate and it ensures the integrity, authenticity and availability of sensed data for the legitimate IoT devices. Moreover, the evaluation of CMA shows an acceptable QoS measurement in terms of computation time overhead, throughput, and packet loss ratio. The future work of this research will focus on developing cryptography mechanism based on encryption and decryption for an IoT network, which will guarantee the confidentiality of the sensed data on IoT networks.

## Figures and Tables

**Table 1:** Emulation Configuration Parameters.

Parameter	Values
MAC and PHY	802.15.14_hmsim and 802.11_hmsim
Propagation Model	Shadowing
Path loss exponent	3.0
Shadowing deviation (dB)	3.0
Emulation area	(1000 m × 1000 m) 1.0 Km^2^
Range of IoT device	150 m
Radio range of BaseST1	250 m
Protocols used	TCP, UDP, ICMP
Number of Intruders	2
Traffic Emulator	Iperf with TCP, Iperf with UDP
Traffic Type	Constant Bit Rate (CBR)
Traffic Load	1 packet/second (pkt/s)–10 packet/second (pkt/s)
Performance metrics	Throughput, latency, packet loss ratio, authentication failure ratio, and computation time overhead
K1, K1 and SALT length size	4 bytes
TOTP	PyOTP
Emulation duration	1000 s
